# Method of predicting Splice Sites based on signal interactions

**DOI:** 10.1186/1745-6150-1-10

**Published:** 2006-04-03

**Authors:** Alexander Churbanov, Igor B Rogozin, Jitender S Deogun, Hesham Ali

**Affiliations:** 1 Department of Computer Science, College of Information Science and Technology, University of Nebraska at Omaha, Omaha, NE68182-0116, USA; 2 NCBI/NLM/NIH, Bldg.38-A, room 5N505A, 8600 Rockville Pike, Bethesda, MD 20894, USA; 3 Department of Computer Science and Engineering, University of Nebraska-Lincoln, Lincoln, NE 68588-0115, USA

## Abstract

**Background:**

Predicting and proper ranking of canonical splice sites (SSs) is a challenging problem in bioinformatics and machine learning communities. Any progress in SSs recognition will lead to better understanding of splicing mechanism. We introduce several new approaches of combining *a priori *knowledge for improved SS detection. First, we design our new Bayesian SS sensor based on oligonucleotide counting. To further enhance prediction quality, we applied our new *de novo *motif detection tool MHMMotif to intronic ends and exons. We combine elements found with sensor information using Naive Bayesian Network, as implemented in our new tool SpliceScan.

**Results:**

According to our tests, the Bayesian sensor outperforms the contemporary Maximum Entropy sensor for 5' SS detection. We report a number of putative Exonic (ESE) and Intronic (ISE) Splicing Enhancers found by MHMMotif tool. T-test statistics on mouse/rat intronic alignments indicates, that detected elements are on average more conserved as compared to other oligos, which supports our assumption of their functional importance. The tool has been shown to outperform the SpliceView, GeneSplicer, NNSplice, Genio and NetUTR tools for the test set of human genes. SpliceScan outperforms all contemporary *ab initio *gene structural prediction tools on the set of 5' UTR gene fragments.

**Conclusion:**

Designed methods have many attractive properties, compared to existing approaches. Bayesian sensor, MHMMotif program and SpliceScan tools are freely available on our web site.

**Reviewers:**

This article was reviewed by Manyuan Long, Arcady Mushegian and Mikhail Gelfand.

## Open peer review

Reviewed by Manyuan Long, Arcady Mushegian and Mikhail Gelfand. For the full reviews, please go to the Reviewers' comments section.

## Background

**Figure 1:**
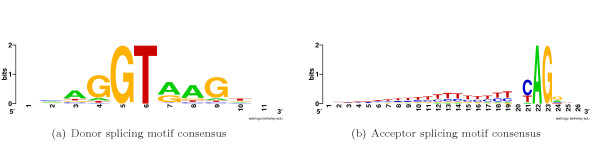
Consensus motifs for donor and acceptor SSs. Y-axis indicates the strength of base composition bias based on information content.

Precise removal of introns from pre-messenger RNAs (pre-mRNAs) by splicing is a critical step in expression of most metazoan genes. The process requires accurate recognition and pairing of 5' and 3' SSs by the splicing machinery. Inappropriate splicing of a gene may result in the translation of a nonfunctional protein. SS motifs are necessary, but not sufficient, for the exact recognition of the exons. Frequently degenerate donor, acceptor and branch point motifs provide insufficient information for exact SS detection [1]. Figure 1 shows SS consensus signals for both 5' and 3' exonic ends. The human transcribed regions have plenty of motifs of unknown functionality with structure very similar to the SS consensus signals (GT or AG dinucleotide surrounded by proper context). These sites are called *splice-like signals *and they outnumber the real sites by several orders of magnitude.

Correct prediction of SSs appears to be the key ingredient in successful *ab initio *gene annotation, since dynamic programming procedures must see all the exon/intron boundaries in order to find the optimal solution [2]. The most sensitive sensor design predicting the least amount of false positives is preferable. Another good feature of a SS sensor is the ability to rank predicted SSs, i.e. to assign a certain score characterizing the importance or strength of a putative site of splicing.

Numerous approaches have been taken towards effective detection of SSs. In our experiments, the highest performance for complete gene structural prediction has been achieved with GenScan [3] and HMMgene [4] tools. Both tools use three-periodicity in coding exons. Codonic composition of coding exons has particular probabilistic properties that allow gene finders to synchronize their prediction engines with gene structure and efficiently stitch exons in frame-consistent fashion [2].

However, all tools relying on three-periodic coding components in their prediction algorithm suffer substantial performance loss if confronted with noncoding exons. On the other hand, the biological splicing process seems to be indifferent to exonic coding potential [5,6]. To alleviate the problem, gene structural prediction tools use information sources directly related to the biological process of splicing [7]. One of the promising mechanisms of SS definition is *signal *interaction, i.e. putative SSs and various ESEs, ISEs in addition to Exonic (ESS) and Intronic (ISS) Splicing Silencers [see Subsection *Splicing signals*].

In this paper we introduce our new gene structural annotation tool SpliceScan. Our tool is based on the Naive Bayesian network that linearly combines the number of splicing-related components to improve SS prediction. Before we describe our tool, we discuss our approach to SS sensor design [see Subsection *Splice Sites sensor*]. We discuss the MHMMotif tool we use to detect putative splicing enhancers [see Subsection De novo *motifs detection*].

### Splicing signals

Specificity in the splicing process derives partly from sequences other than SS signals, including Exonic Splicing Enhancer (ESE) and Exonic Splicing Silencer (ESS) signals [8,9]. ESE signals are required for a constitutive exon definition and for an efficient splicing of weak alternatively spliced exons [10] (while ESS signals suppress the removal of adjacent introns [9,11]), which may lead to exon skipping. There are 10 serine/arginine-rich (SR) Splicing Enhancer proteins known today (SRp20, SC35, SRp46, SRp54, SRp30c, SF2/ASF, SRp40, SRp55, SRp75, 9G8 [12]) and approximately 20 hnRNP Splicing Silencing factors [13], among them the most studied hnRNP A1 complex [11]. Tra2β is reported to be the SR splicing regulator [12]. All the SR proteins have two structural motifs: the RNA Recognition Motif (RRM) binding to certain motifs in RNA; and the arginine/serine-rich (RS) domain responsible for Protein-Protein interactions within splicing complex [12].

Together with inefficient SS signals, the appropriate balance of ESE and ESS elements somehow allows fine tuning of the splicing mechanism [9]. Both 5' U1 snRNP and 3' U2AF^65^·U2AF^35 ^were shown to interact with ESEs [12]. Cross-intron bridging may happen through hnRNP complexes [14]. Experiments show that the 3' end definition is not affected by intron bridging, but is defined solely by the strength of the acceptor site polypyrimidine tract and the position of splicing enhancers and silencers [10,15].

**Figure 2:**
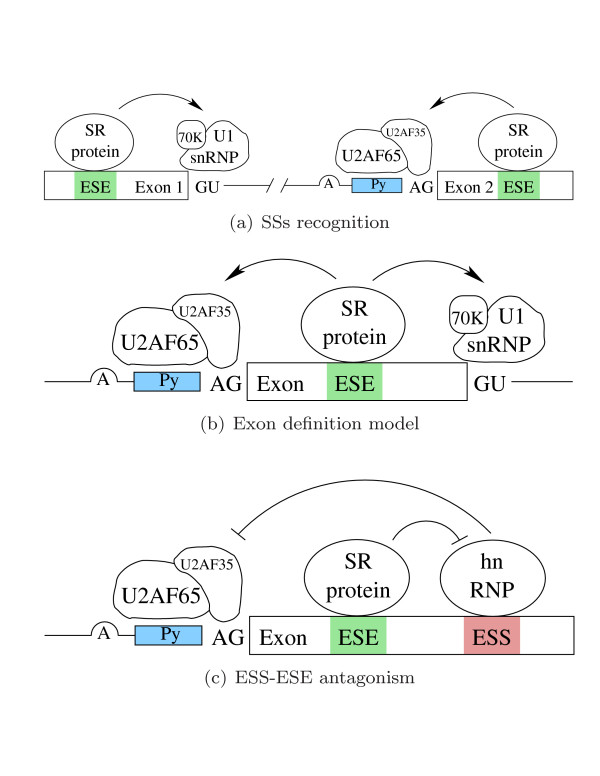
Models of exon definition and ESS-ESE interaction.

The silencing process is still poorly understood [16]. However, there are several models explaining the observed antagonism between hnRNP complexes and SR proteins [17]. For example, hnRNP A1 binds to the ESS and hinders binding of SR proteins to a weak ESE located just downstream of ESS [9]. Several rules have been identified for interaction of ESE/ESS factors with spliceosomal assembly:

• ESE and ESS elements are frequently located in downstream exons [18];

• The precise mechanism by which hnRNP A1 binds the ESS in the upstream exon and represses splicing of the upstream intron remains unknown, although the 3' site is a likely target for repression [9] as shown in Figure 2(c);

• Most splicing enhancers are located within 100 nucleotides of the 3' SS and are not active further away [15];

• Each enhancer complex assembles independently for 3' and 5' sites, and there is a minor interaction across an intron [19], as shown in Figure 2(a);

• Based on current views of exon definition, each exon should be recognized by the splicing machinery as an independent unit [3,19], as shown in Figure 2(b);

• Analysis of the experimental data revealed that the splicing efficiency is directly proportional to the calculated probability of a direct interaction between the enhancer complex and the 3' SS:

- Strong natural enhancers function at a greater distance from the intron than weak natural enhancers do [18];

- The closer an ESE is to a SS, the more efficient it is [15];

- Multiple enhancer sites increase the probability of splicing activation [15];

- Strong ESS sites may suppress an effect from ESE(s) located upstream [9].

To identify known ESE/ESS motifs, we used RRM binding motifs from [20] as shown in Table 1. PolyA signals, that could also be employed by splicing machinery, were detected by oligos reported in [21].

**Table 1:** Nucleotide symbols used: M → (A/C), R → (A/G), W → (A/U), Y → (C/U), S → (C/G), K → (G/U). (Table credit [20])

Protein	High-affinity binding site	Functional ESE
SRp20	WCWWC	GCUCCUCUUCC
	CUCKUCY	CCUCGUCC

SC35	AGSAGAGUA	GRYYMCYR
	GWUWCCUGCUA	UGCYGYY
	GGGUAUGCUG	
	GAGCAGUAGKS	
	GUUCGAGUA	
	UGUUCSAGWU	
	AGGAGAU	

9G8	(GAC)^*n*^	
	ACGAGAGAY	
	WGGACRA	

SF2/ASF	RGAAGAAC	CRSMSGW
	AGGACRRAGC	

SRp40	UGGGAGCRGUYRGCUCGY	YRCRKM

SRp55		YYWCWSG

TRA2β	(GAA)^*n*^	

nhRNP Al	UAGGGW	

## Results and discussion

### Bayesian sensor performance

We use the Receiver Operating Characteristic (ROC) to compare performance of different sensors. A ROC curve is a graphical representation of the tradeoff between the false negative and false positive rates for every possible cutoff. By tradition, the plot shows the false positive rate (1 - *Specificity*) on the *x *axis and the false negative rate (*Sensitivity*) on the *y *axis.

Sensitivity (*Sn*) and Specificity (*Sp*) were calculated according to the formulas





Here *TE *is the number of accurately predicted exon boundaries, *AE *is the number of annotated exon boundaries in the test set and *PE *is the number of predicted exon boundaries.

The accuracy of a test (i.e. the ability of the test to correctly classify cases with a certain condition and cases without the condition) is measured by the area under the ROC curve. An area of 1 represents a perfect test. The closer the curve follows the left-hand and top borders of the ROC space, the more accurate the test; i.e. the true positive rate is high and the false positive rate is low. Statistically, more area under the curve means that it is identifying more true positives while minimizing the number of false positives.

To evaluate Bayesian sensor performance we compiled three test sets:

1. 250 first multiexonic all-canonical SS genes picked from the top of our GIGOgene annotation. This test set includes 2,482 donor and 347,402 donor-like signals in addition to 2,482 acceptor and 465,000 acceptor-like signals.

2. 1,072 human 5' UTR gene-annotated fragments, including the first 50 nt from the CDS region. We picked only GIGOgene annotations containing at least one intron with all canonical SSs. Our 5' UTR test set includes 1,869 donor and 734,744 donor-like signals in addition to 1,846 acceptor and 925,464 acceptor-like signals.

3. 183 rat multiexonic all-canonical SS genes we were able to annotate with GIGOgene. This test set includes 1,405 donor and 240,539 donor-like signals in addition to 1,405 acceptor and 295,640 acceptor-like signals. The test set is specifically included to evaluate cross-specie sensor performance.

**Figure 3:**
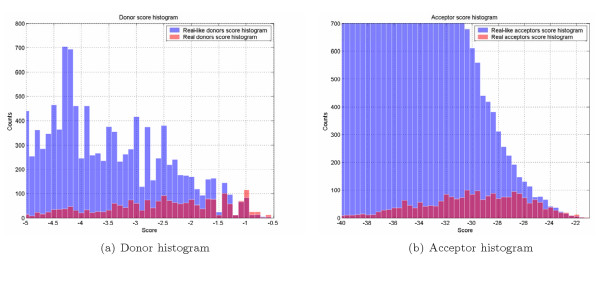
Bayesian sensor histograms produced for 5' SS and 3' SS signals on the test set of 250 human genes.

**Figure 4:**
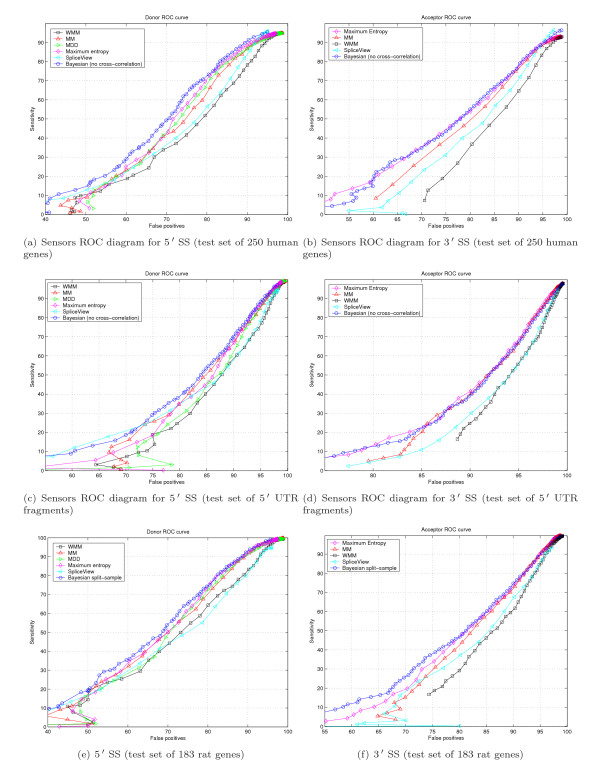
ROC diagrams for Donor and Acceptor signals.

For experimental purposes on human test sets we removed cross-correlating gene-annotated fragments from the learning set. In experiments with human test sets we BLAST-aligned the test set to the learning set and removed all homologous fragments, both human and mouse, with BLASTN hit expected value less than 10^-10 ^and bitscore more than 75 bits. The experimental sensor performance study is shown in Figure 4. ROC curve irregularities could be attributed to multimodal score distribution of splice and splice-like signal, as could be seen in Figure 3. We did not removed cross-correlation between the learning set and the test set of 183 rat genes.

Our Bayesian 5' SS sensor outperforms the recently introduced Maximum Entropy SS sensor [22] and MDD sensor used in GenScan tool [3] as could be seen in Figures 4(a), 4(c) and 4(e). Performance of the Bayesian 3' SS sensor is similar to that of the Maximum Entropy SS sensor [22].

The 5' SS oligonucleotides tend to cluster, only 3,084 nonamers got non-zero probability entries in the sensor table, with the highest 40 ranked motifs shown in Table 2. Among 2,482 true 5' SSs tested in the test set of 250 human genes, 13 nonamers had zero entries in the table which stands for oligonucleotide miss rate of 0.52%. On the other hand, acceptor SSs demonstrates great variability and requires substantially larger learning sets [see Subsection *Learning set size study*].

**Table 2:** Probabilities of being true SS for first 40 statistically most highly ranked putative 5' SS. Probabilities are calculated based on number of times we spot certain nonamer acting as SS as opposed to splice-like signal in our learning set [see Subsection *Splice Sites sensor*]

**Nonamer**	**Probability**	**Spotted as true signal**	**Spotted as true-like signal**
CCG**GT**AAGT	0.701	270	115
CCG**GT**GAGT	0.676	755	361
CGG**GT**AAGT	0.668	270	134
ACG**GT**GAGT	0.625	608	364
CGG**GT**GAGT	0.587	586	412
GCG**GT**AAGT	0.575	184	136
TCG**GT**AAGT	0.568	218	166
CAG**GT**AAGC	0.557	1326	1055
TCG**GT**GAGT	0.556	503	401
ACG**GT**AAGT	0.551	245	200
GCG**GT**GAGT	0.530	569	504
CAG**GT**GAGT	0.523	2561	2335
CAG**GT**AAGT	0.509	1437	1388
AAG**GT**GAGT	0.508	2013	1952
AAG**GT**AAGC	0.504	1057	1039
CGC**GT**AAGT	0.5	36	36
CCG**GT**AAGG	0.489	157	164
CAG**GT**AAGG	0.484	1667	1777
CGG**GT**AAGC	0.482	145	156
AAG**GT**AAGT	0.469	1389	1569
CAG**GT**AAGA	0.469	1917	2172
ACG**GT**AAGG	0.468	162	184
TCG**GT**AAGC	0.457	101	120
CCG**GT**AAGC	0.456	134	160
GAG**GT**AAGT	0.454	978	1175
ACG**GT**ACGT	0.453	29	35
AAG**GT**ACGT	0.452	193	234
AAG**GT**ACGC	0.423	85	116
GAG**GT**GAGT	0.420	1717	2371
AAG**GT**AAGG	0.418	1318	1833
AAG**GT**AAGA	0.416	1610	2261
ACG**GT**AAGC	0.408	118	171
GAG**GT**AAGC	0.408	634	921
TCG**GT**AAGA	0.408	148	215
CCG**GT**AAGA	0.403	160	237
CGG**GT**AAGG	0.397	219	332
ACG**GT**AAGA	0.397	165	251
AAG**GT**GAGC	0.395	1325	2031
CAG**GT**GAGC	0.394	2073	3185
CAG**GT**ACGT	0.392	231	358

Bayesian 3' SS sensor design seems to favor cross-correlation between the learning set and test sequences; cross-correlation removal worsened sensor ROC characteristics [see Subsection *Learning and test sets cross-correlation degree study*]. In reality, sensor performance should be as good or better than reported because we used an extensive set of genes in human and mouse genomes for learning, covering most gene families. Chances are high that a sequence in question substantially cross-correlates with the learning set we use. Test example with 183 rat genes confirms our expectations, as could be seen in Figure 4(f), where ROC of the Bayesian sensor prevails over all other sensor designs.

#### Learning and test sets cross-correlation degree study

**Figure 5:**
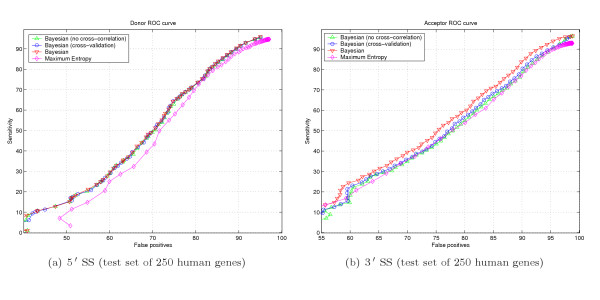
Study of cross-correlation performance dependency.

We study Bayesian sensor performance depending on the degree of cross-correlation between the learning and test sets. Test performance appears to be the best for experiments with intersecting test and learning sets, denoted as "Bayesian" in Figure 5. Removal of test set sequences from the learning set corresponds to curves denoted as "Bayesian (cross-validation)" and further elimination of cross-correlating sequences [see Subsection *Bayesian sensor performance*] corresponds to "Bayesian (no cross-correlation)" curves in Figure 5. Cross-correlation removal has a dramatic effect on the performance of the 3' SS sensor as shown in Figure 5(b). On the other hand, cross-correlation has practically no effect on the performance of the 5' SS sensor as shown in Figure 5(a).

#### Learning set size study

**Figure 6:**
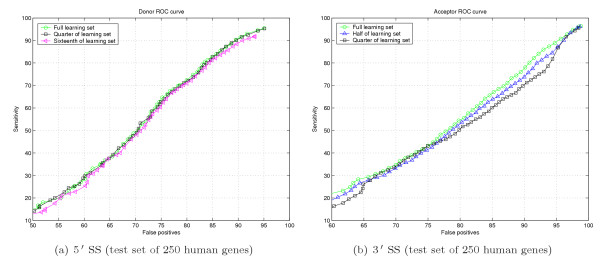
Study of sensor performance based on learning set size.

Our experiments with learning set size indicate, that 5' SS performance tolerates substantial decimation of the learning set without apparent quality loss, as shown in Figure 6(a). Decreased size of learning set causes substantial performance loss for 3' SS sensor, as shown in Figure 6(b).

The learning set we collected [see Subsection *Splice Sites sensor*] is not sufficiently vast for 3' SS sensor to avoid performance loss in case of removed cross-correlation. Ideal 3' SS ROC curve should be similar to the "Bayesian" as shown in Figure 5(b).

### Detection of ISE signals

ISE motifs are essential components for understanding splicing events. In order to predict ISE motifs located in vicinity of 3' and 5' SS, we compiled two sample sets of 2,000 pre-donor and post-acceptor 150 nt fragments known to be real with a high degree of confidence. For fragment extraction we parsed results of GIGOgene [23] spliced alignment of human RefSeq against the phase III human DNA database from NCBI, picking only canonical SSs from predicted multiexonic structures.

**Figure 7:**
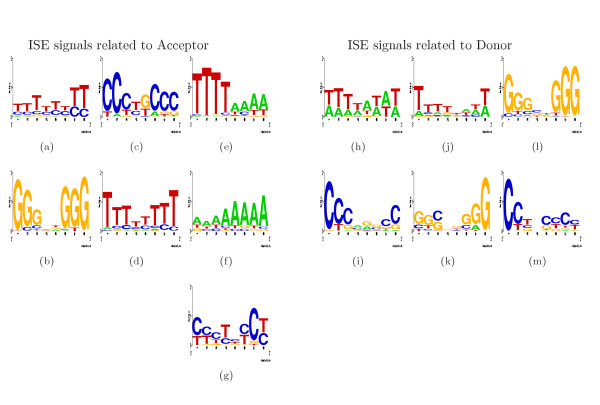
ISE motifs found in vicinity of 3' SS (Figures 7(a)-7(g)) and 5' SS (Figures 7(h)-7(m)).

We applied MHHMotif to these sample sets of sequences, and recovered motifs shown in Figure 7, where representative motifs were obtained from HMM components in "generative mode" [see Additional file 1].

### Intronic conservation study

We adopt conservation criteria to evaluate significance of the putative ISE elements found [see Additional file 1]. We subdivided set of all possible 8-mer oligonucleotides in two subsets: *hypothesis subset*, i.e. all detected putative ISE elements, and *null-hypothesis subset *– the rest of the possible oligos. To study evolutionary conservation we took 3,005 mouse-rat intronic alignments. In our conservation study we considered only first 100 nt and last 100 nt of each alignment, excluding very first 5 nt and very last 5 nt for they playing role in SS highly conserved consensus signals.

We used sliding window of size 8 nucleotides to detect positions matching the set of detected putative ISEs. Ratios of conserved nucleotide positions vs. non-conserved nucleotide positions were calculated for our hypothesis set and null-hypothesis set elements. We removed test pairs where either ISEs were not detected or non of the nucleotides within putative ISEs changed. Alignments too short to extract statistics regions were also removed.

The following probabilities associated with a two-tailed Student's paired t-test were found as shown in Table 3. Detected putative ISE elements are on average more conserved than other oligos. Statistically significant higher evolutionary conservation suggests the biological importance of a significant fraction of these elements in the splicing process.

**Table 3:** T-test probabilities for putative ISE elements found

Putative ISE elements	Test area	Number of alignments	Probability
Donor-related ISEs	Post-donor region	2,544	7.22 × 10^-82^
Donor-related ISEs	Pre-acceptor region	2,586	1.86 × 10^-106^
Acceptor-related ISEs	Pre-acceptor region	2,487	8.39 × 10^-95^
Acceptor-related ISEs	Post-donor region	2,235	6.39 × 10^-59^

### Detecting ESE signals

**Figure 8:**
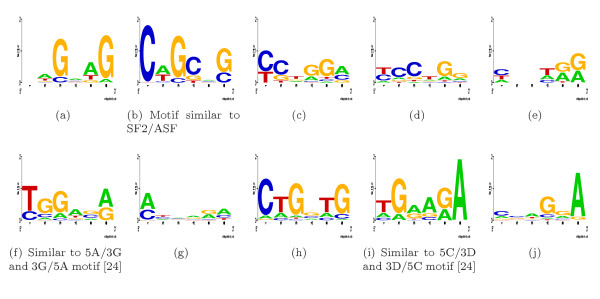
ESE motifs repetitively detected in our MHHMotif runs.

To detect putative ESE signals we applied the MHMMotif tool to the set of 2,000 distinct exons as we parsed the human genome annotation of our GIGOgene tool. In our experiments, motifs in Figures 8(a), 8(b), 8(c), 8(f), and 8(h) converged in two families with similar ESE signal signature but different convolution patterns supporting either 5' or 3' exonic ends. We present putative motifs [see Additional file 1]. Our set of putative ESE signals substantially overlaps with ESEs suggested by Burge and co-workers [24]. Among 202 detected putative ESE elements, 42 are present in this previously reported set of 238 ESEs, which exceeds randomly expected overlap by 3.5 times. Strong evolutionary conservation was found for these ESE signals located near splicing signals [25].

### SpliceScan performance

We use test sets [see Subsection *Bayesian sensor performance*] for the SpliceScan performance estimates. We chose the second test set of non-coding 5' UTRs because it was suggested that at least some introns in 5' UTRs may attenuate translation at the initiation stage [26,27]. Thus, a reliable prediction of introns and strength of splicing signals in 5' UTRs is essential for 5' UTR studies.

**Figure 9:**
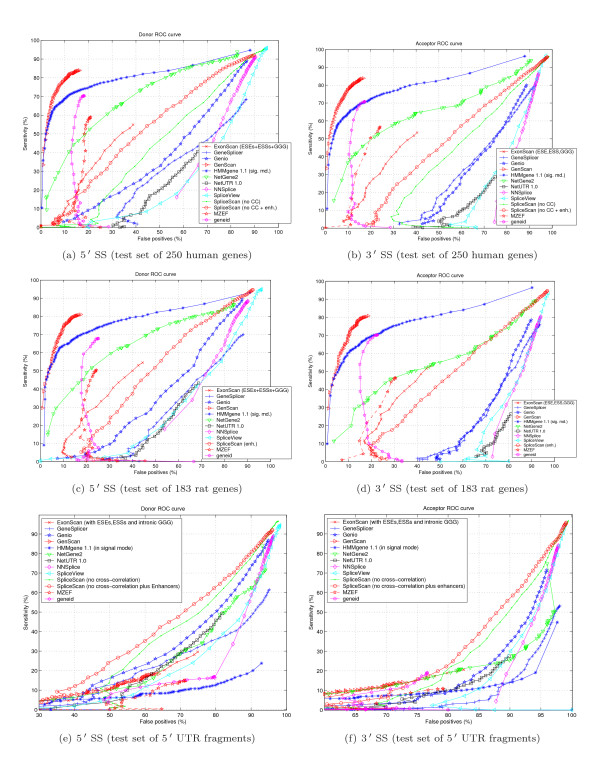
ROC diagrams for Donor and Acceptor applications.

Performance of SpliceScan appears to be the best for short non-coding 5' UTR gene fragments test set, as presented in Figures 9(e) and 9(f). For the test set of 250 human genes and 183 rat genes our tool predicts SSs better than SpliceView [5], GeneSplicer [28], NNSplice [29], Genio [30] and NetUTR [6].

## Conclusion

By trading complexity of sensor design for the size of learning set we were able to substantially improve prediction of the SSs. Bayesian 5' SS sensor demonstrated superior performance, as compared to existing approaches, in all conducted experiments. Neither removal of cross-correlation between the learning and test set, nor fourfold decrease of learning set size were able to compromise the sensor fidelity. Opposite observation were made with 3' SS sensor, where performance is affected both by degree of cross-correlation between learning and test set and the size of the learning set. Bayesian 3' SS sensor demonstrates comparable performance with the Maximum Entropy sensor, when cross-correlation is removed between the learning and test set. The sensor performance improves substantially if we do not specifically remove cross-correlation, as in case of 183 rat genes test set or experiments with the degree of cross-correlation. We believe that performance of our sensor could be generalized to a broad variety of *tetrapoda *organisms; genes encoding splicing RNP complexes are among the most conserved known genes [31].

Using MHMMotif tool we were able to discover motif families for ESE/ISE elements. Small fraction of our detected putative ESE elements correspond to previously reported ESE motifs [24], other elements could be considered as novel. Statistically significant average conservation ratio for putative ISE elements, as compared to other motifs, supports their functional importance in human genome.

Our predicted ISE and ESE elements have substantial impact on splicing, as we were able to improve SS prediction using these elements. Based on linear model of splicing factors interaction, SpliceScan has been able to outperform SpliceView [5], GeneSplicer [28], NNSplice [29], Genio [30] and NetUTR [6] tools in all the test categories.

SpliceScan did not to perform better than GenScan [3], HMMgene [4], NetGene2 [32,33], MZEF [34], Geneid [35,36] and ExonScan [7] on the test set 250 human genes and 183 rat genes. The reason is that SpliceScan does not rely on three periodicity property to discover the coding exons. ExonScan, which uses exon definition model combined with ESS/ESE elements for better prediction quality, is another tool implemented as splicing simulator with objectives similar to SpliceScan. It has the same "average" ROC profile for not using exonic coding potential statistics. Exon definition model of ExonScan seems to work better for internal boundaries prediction, but suffers in case of predicting boundaries of incomplete exons, first and last exons, as all the other tools do.

In our experiments on the human test sets we removed cross-correlation from the learning set, that made performance of our tool worse as it would normally be. Since we do not have control over the learning sets of the competing tools, performance of these tools is likely to be positively affected by overlaps between their learning sets and our test sets. The test set of 183 rat genes exemplifies the performance issues with the existing tools that were trained on the human learning sets; they usually perform worse when confronted with new sequences. ExonScan does not seem to lose prediction quality when confronted with sequences from other closely related organisms.

SpliceScan performs best on the set 5' UTR fragments because of the *SS definition *model we use, i.e. we combine all available information for a certain SS without mandatory requirement of large contexts or having other corresponding exonic boundary.

Bayesian sensor, MHMMotif program and SpliceScan tools are freely available on our web site [37].

## Methods

### Splice Sites sensor

SSs are known to contain complex interdependencies between nonadjacent positions that could be attributed to evolutionary pressure from very complex biological splicing machinery. Recovery of these interdependencies is difficult problem in machine learning, but may lead to better understanding of the splicing process and improved sensor design.

Numerous approaches have been taken towards effective detection of SS [38-50]. The earliest SS sensors were the Weight Matrix Model (WMM) [51], Weight Array Model (WAM) [52], and Windowed second-order WAM model (WWAM) [3]. The Maximal Dependence Decomposition (MDD) sensor mentioned in [3] outperformed previously known 5' SS sensors. It explores long-range dependencies in the donor 5' motif by iterative subdivision at each stage splitting on the most dependent position, suitably denned. Leaves of the resulting bifurcation tree appear to be simple WMM models. Another approach, published in parallel and implemented in the SpliceView program [5], explores the same idea of creating WMM motif families with a clustering algorithm, which leads to better performance compared to simple WMM and WAM.

Various sensors were built later or in parallel based on Bayesian Networks [53,54], Neural Networks [29,55] and Boltzmann machine with Bahadur expansion [56]. Also there was a recent study based on Support Vector Machine (SVM) [57]. None of these methods were shown to outperform MDD for 5' SSs. A new Maximum Entropy sensor [22], Permuted Markov Models [58] and approach based on dependency graphs and their expanded Bayesian network [59] outperformed MDD on 5' SSs.

Methods that use subdivision of SSs into families of consensus motifs, such as MDD, SpliceView, Maximum Entropy sensor and Permuted Markov Models, appear to have the highest performance as they potentially can reveal distant correlations by putting similar-looking motifs into the same probabilistic class with its own consensus.

**Figure 10:**
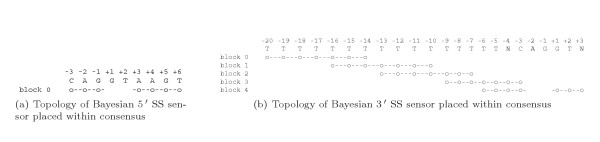
Blocks placement within consensus.

We use a simpler sensor design based on 7-mer oligonucleotide counting (16,384 possible oligos) in splice and splice-like signals. We place 7-mer blocks within SS consensus signals similar to Maximum Entropy Sensor [22,60], as shown in Figure 10.

We used our GIGOgene [61] tool to collect an extensive learning set of predicted human and mouse gene structures from which we extracted 179,079 donor and 34,258,282 donor-like signals (surrounding GT dinucleotide) plus 179,076 acceptor and 44,353,884 acceptor-like signals (surrounding AG dinucleotide). Based on collected oligonucleotide frequencies, we can evaluate the probability of a 5' SS given an oligonucleotide (GT surrounded by a context)



where *P*(*ss*) – prior probability of an oligonucleotide to be 5' SS, *P*(¬*ss*) – prior probability of an oligonucleotide to be donor-like signal, *P*(*oligo*|*ss*) – likelihood of oligonucleotide in case of 5' SS, *P*(*oligo*|¬*ss*) – likelihood of oligonucleotide in case of donor-like signal. With the donor sensor we output logarithm of the block 0, given certain oligonucleotide, while in case of acceptor sensor we output sum of probability logarithms for all blocks that are calculated with formula similar to (3) under 3' SS condition. An advantage of our sensor design, compared to other projects, is in use of an extensive learning set. Because of the simple sensor structure we can combine very large numbers of splice and splice-like signals in the learning set without a dramatic learning time increase, which substantially improves sensor performance [see Subsection *Bayesian sensor performance*]. Close homologs or repeating gene structures in the learning set do not affect the resulting Bayesian SS sensor prediction quality; repeating domains will equally contribute scores to nominator and denominator in (3) with the resulting Bayesian probability staying the same. Our learning set is *well-balanced*, i.e. we use naturally occurring signals to calculate proper signal/noise ratios.

### De novo motifs detection

Detection and explanation of subtle motifs in human genes is very important, since many mutations disrupting these elements may affect gene transcription, splicing or translation with possible severe consequences [62]. The problem could be formulated as a standard missing-value inference and model parameter estimation. Many approaches of motif detection are based on standard machine learning techniques, such as *Gibbs sampling *and *Expectation Maximization *(EM) algorithms. Among the tools using these algorithms are MEME [63], AlignACE [64], LOGOS [65], BioProspector [66], Gibbs Motif Sampler [67] and many others. Different approaches explore the idea of word enumeration, dictionaries and string clustering, for example RESCUE-ESE technique [24] and a recent method based on probabilistic suffix trees [68]. An interesting method of using prior knowledge in motif finding process has been presented in the LOGOS framework [65], where specific knowledge of DNA-specific bell- and U-shaped motifs signatures has been incorporated. The RESCUE-ESE method [69] is based on a prior belief that ESEs preferentially support weak SSs.

In design of our application we were primarily interested in use of prior knowledge to detect constitutive splicing enhancing elements. MHMMotif is designed to search for motifs in pre-mRNA, the single-stranded molecule. Only a fraction of sequences are assumed to have motifs of certain type within them. In our experiments we saw no apparent correlation between mRNA factors, so we assume that motifs are colocalized independently at a certain distance from target sites (e.g. TSS or SS). We also assume that motifs come in a localized *family*, and many of them are highly degenerate. Furthermore, motif families could be subdivided into *subfamilies*, with overall prediction quality improving.

**Figure 11:**
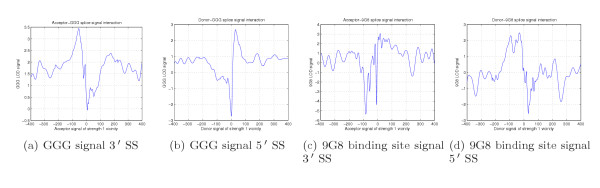
LOD diagram for GGG signal, reported as an ISE (Figures 11(a), 11(b)). LOD diagram for 9G8 signals, reported as an ESE (Figures 11(c),11(d))

As an example of preferential motif location, we present in Figure 11 Logarithm of Odds (LOD) diagrams we measured in the vicinity of SSs for two known ISE and ESE motifs, GGG [70] and (GAC)^*n*^/ACGAGAGAY/WGGACRA (9G8 binding site) [62]. We calculate LOD diagrams as the logarithm of the ratio of signal concentration near SS to the signal concentration near splice-like signal [see Section *Using detected signals to improve SS prediction*]. The signals have distinct bell-shaped concentration increase or decrease once we are getting closer to SS.

Our signal-detecting framework is purely based on Hidden Markov Models (HMM). HMM has many attractive properties in the context of motif detection, such as a tractable probabilistic inference and learning procedure, flexible topology, and ability to incorporate prior knowledge.

### Using the mixture of Hidden Markov Models

**Figure 12:**
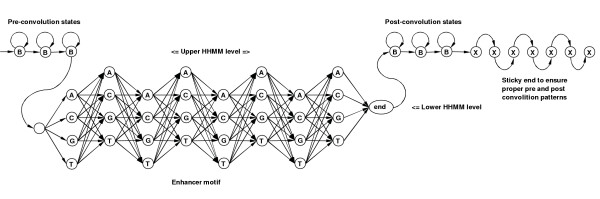
In our HHMM motif model B denotes background state – equiprobable emission of A, C, G, T. X is a special marker for sticky end handling to ensure proper convolution patterns. Sticky end of 10 X's is automatically added to every sample sequence by our tool.

Our strategy of motif detection is based on clustering with Mixture of Hidden Markov Models (MHMM). MHMM has a long record of successful implementations that started in speech recognition [71] and later were used for clustering protein families [72] and sequences [73]. To simulate location constraints of motifs within mRNA we use *convolution *of geometric states, that is described with bell-shaped *negative binomial distribution*, characterized by parameters *p *(probability to stay in the same state) and *n *(number of consecutive states in convolution) [74]. To detect motifs we fit our MHMM model, shown in Figure 12, to the set of sample sequences, using the Baum-Welch algorithm described in [75,76]. The mixture component, shown in Figure 12, is a Hierarchical HMM (HHMM) with stack transformation to a plain HMM, as described in [77].

Many contemporary motif finders [63,67] use *Product Multinomial *(PM) model [65]. PM model corresponds to the widely known binding motif *consensus*, which could be easily visualized with *logos *[78]. The HMM motif model is strictly more general than the standard PM model, since the HMM model is capable of catching dependencies between adjacent motif positions, which used to be a major improvement to weight matrices (PM model) [52]. Furthermore, a mixture of HMMs is potentially able to recognize dependencies between nonadjacent positions, by subdividing motif families into several subfamilies, each catching a specific group of dependencies between local positions.

**Figure 13:**
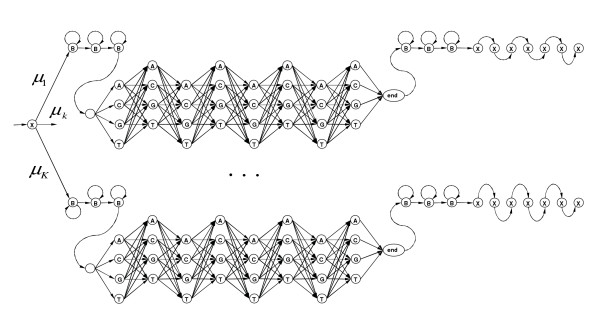
MHMM model we use, where μ_1_, ..., μ_*K *_are the mixing proportions of components such that .

By implementing the MHMM topology, as shown in Figure 13, we simultaneously learn competing HMM components of the mixture, so that they pick various competing motifs. Our application MHMMotif picks signals even if their observation frequency is low compared to simple background, i.e. a statistically significant concentration of a certain signal is not a primary detection criteria. Internal signal structure and its preferential location are the main conditions for our tool. This is another advantage of our approach compared to existing methods.

Since prediction of SS enhancing elements is still an active research topic, we decided to supplement the known enhancing motifs [see Subsection *Splicing signals*] with newly discovered putative enhancers. Our initial assumptions of motif finding capability of MHMM were supported by successful preliminary test results on artificial data sets, where known SF2/ASF [79] and SC35 [80] elements were detected against random background. Another experiment on the sample set of 1,871 human promoter segments (-150...+10 bps relative to initial codon) from The Eukaryotic Promoter Database [81], clearly identified known landmarks of this area, such as OCT-1, NF-1 and AP-2 factors in addition to TATA-box, CAT-box, GC-box and TATA-like A-box factor. Having preliminary-encouraging results, we applied MHMMotif tool to the splicing data sets [see Subsections *Detection of ISE signals *and *Detecting ESE signals*].

### Using detected signals to improve SS prediction

Found ESE and ISE motifs have been evaluated for the ability to improve SS prediction with our new splicing simulator SpliceScan. Our splicing model is based on various-strength SS interaction with signals, such as SS themselves, and Enhancing/Silencing motifs located nearby.

SS classification enhancement in our system follows Bayesian rule in terms of Logarithm of ODds ratio (LOD) [82]



The quantity *Prob*(*D*|*H*) is called the likelihood of the data *D *(in our case ISE, ISS, ESE, ESS and competing SS signals) under hypothesis *H*. The last term of (4) is the LOD ratio of the prior probabilities of the Splice versus Splice-like signals, obtained with the Bayesian sensor. The first sum term in (4) takes into account the evidence provided by the data and comes up with a valid posterior LOD ratio. In order to make a noise-tolerant conversion of donor and acceptor probabilistic sensor scores into prior LOD, we approximate real signal score distribution histograms with a mixture of Beta Probability Density Functions (PDFs). The PDF of the *Beta distribution *is



and the mixture of *n *components is



**Figure 14:**
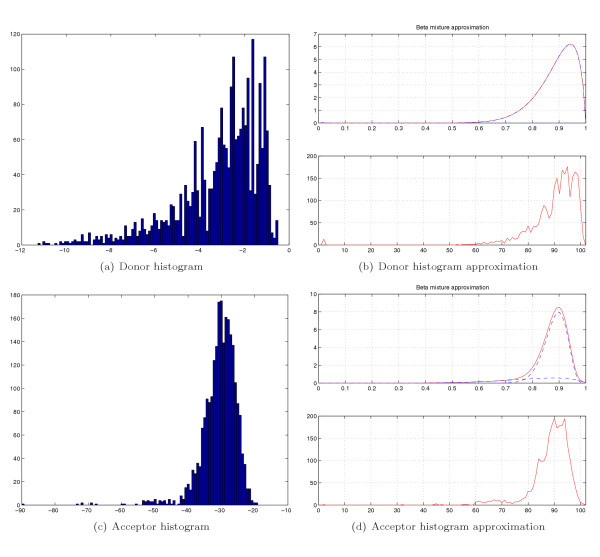
Donor and acceptor histograms approximated with a mixture of Beta distributions.

. Using the Expectation Maximization algorithm for mixture learning, as explained in [83], we fitted the beta mixture (5) to the donor and acceptor SS score histograms as shown in Figure 14.

The donor histogram (shown in Figure 14(a)) was fitted in the range from -25.1 to -0.38 with the mixture

*Mix*_*donor*_(*p*) = 0.006 × *Beta*(*p*, 0.06, 0.91) + 0.994 × *Beta*(*p*, 14.27, 1.80)

and the acceptor histogram (shown in Figure 14(c)) was fitted in the range from -111.01 to -19.19 with the mixture

*Mix*_*acceptor *_(*p*) = 0.83 × *Beta*(*p*, 48.42, 6.37) + 0.17 × *Beta*(*p*, 7.18, 1.85)

where we assume probability argument in the formula



We then calculated the Cumulative Distribution Function (CDF) and subdivided the result probability into 10 equal intervals with corresponding average LOD scores as shown in Table 4.

**Table 4:** Prior LODs

Strength	**Probability**	**Prior donor LOD**	**Prior acceptor LOD**
1	0–0.1	-6.89	-9.10
2	0.1–0.2	-3.53	-5.65
3	0.2–0.3	-2.54	-4.51
4	0.3–0.4	-1.41	-3.69
5	0.4–0.5	-0.50	-2.84
6	0.5–0.6	0.01	-2.38
7	0.6–0.7	0.60	-1.86
8	0.7–0.8	1.06	-0.89
9	0.8–0.9	1.99	-0.26
10	0.9–1.0	1.93	0.88

**Figure 15:**
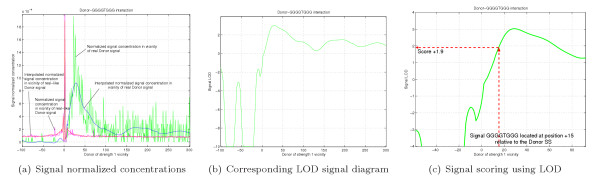
Example of ISE signal interactions.

We built LOD diagram for each of the enhancing motif interacting with SS of different strengths (in range from 1 to 10), similar to shown in Figure 15. First, we measure normalized signal concentrations around SS, as shown in Figure 15(a). Using Matlab^® ^polynomial interpolation we approximated characteristics as could be seen in Figure 15(a). In order to find LOD characteristic, shown in Figure 15(b), we calculate , where *Prob*(*D*|*H*_*SS*_) is normalized signal concentration at certain location next to a SS and *Prob*(*D*|*H*_¬*SS*_) is normalized signal concentration at certain location next to a splice-like signal. Signal LOD scoring happens as schematically shown in Figure 15(c).

We were not able to use all the available enhancing/silencing signals for SS prediction. Part of the problem appears that the linear LOD sum accumulates noise and can only include limited number of factors. The signals we used for donor enhancement are all ISE elements supporting 5' SS shown in 7(h)-7(m), plus ESE element shown in Figure 8(a), 8(d), 8(g) and 8(j) combined with hnRNP A1, poly A and srp20 signals [see Subsection *Splicing signals*]. Surprisingly, polyadenylation signal use contributed substantial enhancement to the ROC diagram. We were not able to use SF2/ASF and SC35 enhancing signals for having LOD characteristics with indistinct enhancement profile. For acceptor enhancement we used ISE elements supporting 3' SS shown in Figures 7(b), 7(c) and 7(e) plus ESE element shown in Figure 8(a), 8(d) and 8(g) combined with hnRNPAl, polyA and srp20 signals.

**Figure 16:**
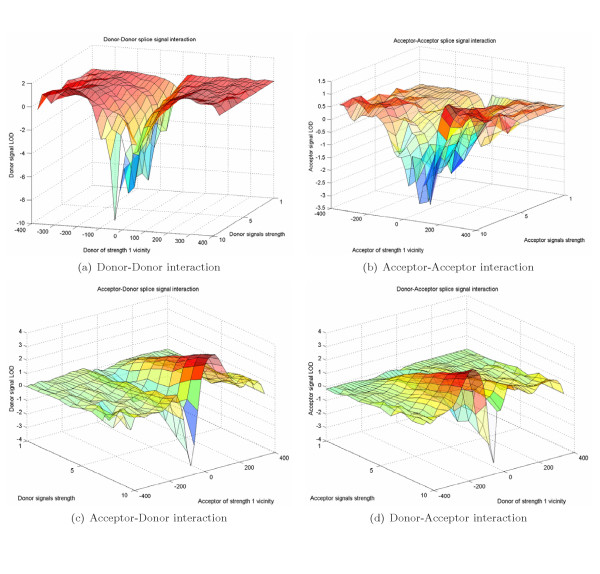
LOD diagrams for Donor and Acceptor signal interactions.

SS/SS interaction LOD diagrams are the key ingredient of our prediction algorithm as they contribute most to SS prediction enhancement. Figure 16 shows various interaction diagrams. X-axis is the SS vicinity position, where SS of strength 1 located at 0, Y-axis is the strength of interacting signal and Z-axis is the LOD characteristic. The main conclusion we make based in Figures 16(a) and 16(b) is that weak signal preferentially avoids strong competitors nearby, especially inside exon, as they can redefine exonic boundary. Figures 16(c) and 16(d) indicate that weak signal (donor or acceptor) has preferential need for strong complementing exonic boundary.

## Authors' contributions

AC, HA and IR conceptualized the project. AC implemented, learned and tested Bayesian sensor, SpliceScan and MHMMotif. HA and JD helped to design the SpliceScan and MHMMotif tools, provided general support. AC wrote the manuscript. All authors read and approved the final manuscript.

## Reviewers' comments

### Reviewer's report 1

Manyuan Long, Department of Ecology and Evolution, The University of Chicago Chicago, United States, Email: mlong@uchicago.edu

The authors attempted to develop a simple sensor to detect splice and splice- site signals. A 7-mer was designed to scan a sequence. The ROC diagrams (Fig. 3) showed its obvious advantage, significantly higher specificity and sensitivity than other methods. In addition, the authors also used MHMM to detect ISE and ESE signals and used found signals to improve SS prediction. I think that the authors developed useful new methods for SS detection and I favor its publication in Biology Direct. However, I also have following minor concerns and hope them get fixed in revision.

Page 1: "Figure 1...": is not original, some sources should be cited (for example, the early work of Tom Schnider of NCI in 1992?". Originality is something that a paper in bioinformatics wants to emphasize.

#### Author response

*For graphical representation of splicing motif consensuses we extracted multiple splicing motifs from our database and used WebLogo tool *[78]*to build the logos*

Page 1: " the human transcribed region have plenty of motif....": it should be pointed out how are these motifs defined and why mentioned here? Is it relevant to the intron splicing?

#### Author response

*Many oligonucleotides have composition identical to known potent splicing signals and at the same time are not supported by spliced alignment*. Ab initio *SSs prediction has to filter out such signals to predict the correct gene structure(s)*.

Page 2, the second paragraph, the caveat of current methods to detect SSs is pointed out: non-coding exons do not have three-periodic coding components. The idea used is the signal interaction: SSs, ISE, ESE, ESS and ISS. New gene structural annotation tool SliceScan is developed and reported in this paper. SS sensor is the key and several majors SS sensors reviewed.

Page 3: in the proposal of a new sensor and compute P (7-mer and SS), why to choose the 7-mer rather than 8-mer or 6-mer should be explained. In addition, the sign – I guess is "non-ss" should be defined. If my guess is correct, this equation makes sense. Biology Direct is a journal for general biology audience; not only for computational biologist so the jargons and special signs should be avoided or if having to use them, explanations should be given.

#### Author response

*7-mer is the size of donor consensus minus GT dinucleotide, since it is always the same, as could be seen in Figure *1(a). *For the modelling of acceptor signal 7-mer appears to be optimal: shorter oligonucleotide will have limited capability of representing long-range positional correlations, while longer oligonucleotides will produce large combinatorial table difficult to learn*.

Page 7: I am not sure how they identify ISEs; section 3.2 is unclear. It seems the conservation is the only criterion. This might be reasonable in a narrow scale of evolution. But given the high evolutionary rate of intron sequence with a lot insertion-deletion (indels), I am suspicious of its feasibility because of the difficulty in alignments to identify the short homologous sequences. Although I do not oppose the approach, a cautionary note in the discussion should be given, which I think will be useful to colleagues.

#### Author response

*ISE signals are predicted using EM learning of MHMM model on intronic fragments of human genes. Main detection criteria used*:

*1. Close localization of putative signals to the intronic boundaries*,

*2. Constant size of putative enhancer*,

*3. Affinity of putative enhancing element to a certain HMM profile*.

*To test hypothesis of their higher conservation, compared to other oligonucleotides, we use mouse-rat intronic alignments that have substantial conserved domains*.

Typo: Page 12: should be put in a different place to make reference continuous.

### Reviewer's report 2

Arcady Mushegian, Stowers Institute, Kansas City, United States

Email: ARM@Stowers-Institute.org

Good: 1. Part 2: the main idea appears to be to trade a more complex model for a larger training set. This seems to improve specificity of the splicing site detection.

Two relevant issues that are not discussed but should be: a. In Figure 3, all ROC curves are still below the non-discrimination line – is this acceptable.

#### Author response

*We use ROC curve different from common True Positive Fraction vs. False Positive Fraction plot, where diagonal is a non-discriminant test result. In our test we know total number of positive cases, so we can build Sn vs. 1 - Sp curve, which is more informative for application comparison purposes*.

b. Gain of the current method is more evident in the lower FP zone, where sensitivity is also low.

#### Author response

*All application ROC curves converge to one point with 100% sensitivity and 0% specificity. The curves differ for lower sensitivity values, where we can speculate about prediction quality. Some applications, like NetUTR and ExonScan, have sensitivity artificially limited to *~50%. Performance analysis for such applications makes sense only in lower sensitivity quarters.

2. A repertoire of intronic splicing enhancers was detected, which is interesting. Not so good: 1. Very unclear writing at different levels:

a. Various inconsistencies and poorly defined terms, for example on pg. 3–4, authors say that they compiled two test sets, and then describe three. Or on pg. 4, line 8 and further: what is "cross-correlating"?

#### Author response

*Cross-correlation means the genes in learning and test set have extensive homologous regions, which favorably affects sensor performance on the test set and should be avoided for rigorous comparison*.

b. section 3.1 : MHMM is not described well: we see a mix of introductory references on general HMMs, of more specialized references that may be telling something relevant but we do not know that, and cat's cradle pictures which are not self-explanatory (and what about these mu parameters?).

#### Author response

*Here we try to reach reasonable compromise between complete system definition and skipping details of well known results from artificial intelligence community, which we reference. Please refer to MHMMotif application source code for more details*.

2. The Results section mentions the programs that work less well than SpliceScan. But we do not hear about comparison between SpliceScan (which barely gets over the non-discrimination line) and half a dozen other, more successful methods represented on the same plots. If the goal of the work was to improve the ab initio approach (cf a line in the abstract), this has to be maintained as the message throughout the paper.

#### Author response

*Our method has clear advantage in case of *5' *UTR gene fragment structural prediction according to ROC curves shown in Figures *9(e)*and *9(f). *In case of gene structural prediction in CDS area, one should use different application, such as GenScan, since SpliceScan does not have frame-consistent synchronization component*.

Overall, this manuscript reads more like the technical report on the ongoing project than a stand-alone paper.

I declare that I have no competing interests.

### Reviewer's report 3

Mikhail Gelfand, Institute of Information Transfer Problems, Moscow, Russian Federation Email: gelfand@iitp.ru

On "Method of predicting splice sites based on signal interactions" by A.Tchourbanov et al., submitted to "Biology Direct"

The problem of identification of donor and acceptor splicing sites is not new, but far from solved, whereas identification of sites regulating splicing (exonic/intronic splicing enhancers/silencers) has emerged relatively recently. Given the importance of both these problems for gene recognition and understanding alternative splicing, any progress in this area is most welcome.

The authors attempt to address both problems in one framework of Bayesian analysis. They apply Bayesian sensors to detection of donor and acceptor splicing sites. The exposition in this part (section 2, pp. 2–3) contains several gaps. It is not clear how well the described approach of 7-mer counting with subsequent Bayesian weighting generalizes; in particular, it seems that the sensors will not accept a completely new 7-mer as a site. If the authors implicitly claim that all possible 7-mers have already been observed in the training set, and the only problem is proper weighting, this needs to be substantiated. A helpful piece of data would be the rank distribution of 7-mers in the positive and negative sets. How many 7-mers have been observed only once in the positive set (and would be missed if only half of that set were used for training)?

#### Author response

*With cross correlation removed between learning set and the test set, when testing on the set of 250 human genes, we had miss rate of 0.52% for our *5'*SS sensor, which is acceptably low value. For *3'*SS sensor overall miss rate is negligibly low, since sensor topology is composite of several blocks. We show top 40 ranking *5'*SS nonamers in Table *2. *We added discussion on sensor performance related to learning set size [see Subsection *Learning set size study/, *where we show that Bayesian sensor has preference for the large learning sets. For example, the sensor could be successfully applied to recognition of the Translation Initiation Site (TIS) against upstream A UGs, where we can collect large learning set. In the TIS sensor design we used three strategically located heptamers, so that they can catch both long-range dependencies and initial codon bias, as shown below:*



*We used 42,883 TIS and 77,140 TIS-like signals from human, mouse and rat RefSeq databases to learn our TIS Bayesian sensor, which demonstrated, in our preliminary experiments, superior performance as compared to simple Kozak's consensus rule (GCC)GCCRCCAUGG (where R = G or A) *[84]* and corresponding weight matrix. However, the sensor design does not generalize well to recognition of other signal types, such as transcription factors, with very thin learning sets*. 

Another missing part of exposition is a formula for combining several sensors for acceptor site analysis. Is the final score (probability) obtained by multiplying probabilities assigned by the sensors?

#### Author response

*Acceptor sensor uses product of block probabilities*.

Given the possibility of over-fitting, the testing procedure should be designed very carefully. Description in section 2.1 (pp. 3–4) does not address several issues, the most important of which is the influence of homologous sites in the training and testing data. The authors mention that they have removed homologs from the human sets, but it is not clear whether only human paralogs have been considered, or mouse homologs as well. It is not clear also whether the rat set has been purged from homologs to sites used in training. A minor note is that the text (end of p.3) mentions two datasets, whereas three sets are listed.

The authors completely ignore the problem of alternative splicing.

#### Author response

*Both human and mouse homologs were removed from the learning set in our experiments. Domains, paralogous to the rat test set, were not been specifically purged from the learning set. In the case of rat test set we were interested in the performance test on similar, but substantially diverged organism, i.e. simulation of practical sensor application. We considered prediction of genomic structures the way they are annotated in GenBank. Indeed, some of the predicted SSs could be alternatively committed, but this is another topic for study*.

The behavior of ROC curves (Fig. 3) seems to be somewhat erratic. In particular, they are not even monotonic. Probably that means that the distribution of scores on positive and negative sets is not unimodal. Anyhow, these distributions should be presented in addition to the ROC curve data. On a technical side, it would be most helpful if the data were plotted using uniform scales; otherwise it is difficult to compare curves on different plots. The authors should also explain how they produced ROC curves for other methods: whether they had been re-programmed or some existing programs (stand- alone or internet servers) were used, what versions, etc. Otherwise these pieces of data are not easily reproducible.

#### Author response

*The distribution of scores returned by different methods is multimodal, as shown in Figures *3(a)*and *3(b). *We used large number of possible intermediate points to reproduce fine features of the curves and to avoid possible graph extrapolation between distant points. The curves were obtained using Java web application, which sends queries of genomic structures to different online tools, collects statistics and outputs data points for ROC curves reconstruction*. 

The last sections of the manuscript are somewhat fuzzy. The authors identify a number of likely splicing enhancers/silencers, and then use these signals to improve site detection (section 4). However, is absolutely is not clear, how this improvement is implemented, nor whether the results become stronger: the entire "Results" section (4.1) consists of two short paragraphs and a huge figure featuring the ROC curves. The test sets are not described: what portions of adjacent exons and/or introns were considered? Again, the behavior of the ROC curves in many cases looks absolutely erratic: they are convex, concave, and even zigzagging. The reasons for that are not discussed.

#### Author response

*Results of SpliceScan become much stronger compare to simple Bayesian SS sensor. In our algorithm we try to guess the boundaries of region eligible for LOD scoring by looking at the surrounding putative complementing SSs. For example, for *5' *SS we consider the nearest *3' *SS downstream as the beginning of next exon, and the first upstream *3' *SS as the opposite side exonic boundary. Weak signals are abundant, which results in unnecessarily tight region boundaries. By relaxing requirements for the region boundary candidates to be stronger than 1, we substantially extend region boundaries and count additional enhancing signals, which improves performance. However, further relaxing of boundaries will put many signals in the wrong spot (signals that assumed to be within intron region might reside in exons with corresponding LOD score miscalculation), which worsens the ROC characteristic. The maximum allowed distance of the region expansion is *-200...+ 300 *bp for the *5' *SS and *-300...+ 200 *bp for the *3' SS. *Many applications tend to produce multimodal score distributions for the splice and splice-like signals, which causes ROC curves jitter*.

Minor remarks.

" P. 2. Splicing silencers are mentioned in the introduction, but not addressed during analysis. At that, it is not clear how do the authors assign activation/repression function to their identified motifs: they could well function as silencers.

" P. 7. The claim that ISEs have never been systematically analyzed (section 3.2) is not correct.

" P. 7. What are the definitions of parameters in the formula (conserved/non-conserved)?

" P. 9. The first sentence in the last paragraph on this page is obscure. What are "SS of different strengths"? That is, what groups of sites, or what strength intervals, or whatever have been used?

" P. 9. Definition of D: is it a competing SS or a splicing enhancer?

" Ref. 9 = Ref. 11.

" Use of capitals in the reference list is erratic. "DNA", "Markov", "Bayesian" need consistent capitals.

Overall, I believe that, although the study has produced some interesting observations, and the authors' approach seems promising, the manuscript in the present form is rather raw and badly structured (it really looks like several independent papers half-written and stitched together), and several important points are not addressed at all.

I declare that I have no competing interests.

## Supplementary Material

Additional File 1 The file includes detected ESE/ISE consensus motif logos along with oligonucleotides generated by components of MHMM. Click here for file
