# Supplementary material for: Method of predicting Splice Sites based on signal interactions
Source: Biol Direct. 2006 Apr 3;1:10. doi: 10.1186/1745-6150-1-10 (PMC1526722; doi:10.1186/1745-6150-1-10)
Supplement: Additional File 1 — The file includes detected ESE/ISE consensus motif logos along with oligonucleotides generated by components of MHMM. [file 1745-6150-1-10-S1.pdf]

Supplemental materials

1 Detected putative enhancing motifs

1.1 Acceptor ISE elements

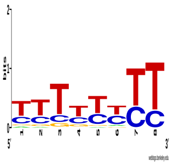

CCTTTCTT, CTTCTCTT, CTTTCCTT, CTTTCTTT, TCTGTCTT, TTCCTCTT, TTCCTTTT, TTGCTCTT, TTTCCCTT, TTTCTCTT, TTTGTATT, TTTTCATT, TTTTCCTT, TTTTCTCC

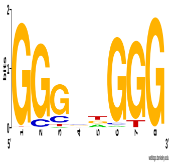

GAAGTGGG, GCTCAGGG, GCTCTGGG, GCTTTGGG, GGGACCAA, GGGACCCT, GGGAGAAA, GGGAGAGG, GGGAGATG, GGGAGGGG, GGGGTGGG, TTCCAGGG, TTCCTGGG

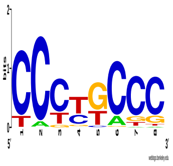

CAGGCACC, CAGGCAGG, CAGGGCCC, CCCAGCCC, CCCCTCCC, CCCCTCCT, CCCTGACC, CCCTGAGG, CCCTGCCC, CCCTGCCT, CCCTGCTC, CCCTGCTG, CCCTGGAA, CCTCTCCC, CCTCTCTC, CCTCTCTG, TCCAGCCC, TCCCTCCC, TCCTGAGG, TCCTGCCC, TCCTGCCT, TCTCTCCC

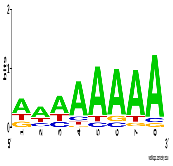

AAAAAAAA, AAAGAAAA, AGAAAAAA, GCCCTGGA, GCCCTGGG, GCCCTGTC, GCCCTGTG, GCCTCCCA, GCCTCCTC, GCCTCCTG, GGAAAAAA, GGAGAAAA, TTTAAAAA

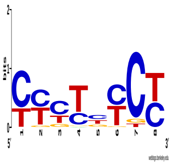

CCCTCCCC, CCCTCCCT, CCCTCTCC, CCCTCTCT, CCCTGTCT, CCCTTCCC, CCTCTCCT, CCTCTTCC, CCTCTTCT, CCTTCCCC, CCTTCTCC, CCTTCTCT, CCTTGCCC, CCTTGTCC, CTCCACCC, CTCCTCCC, CTCCTTCC, CTCTCCCC, CTCTCTCC, CTCTCTCT, CTCTGCCC, CTCTGCCT, CTCTGTCC, CTCTGTCT, CTCTTCCC, CTGTCCCC, CTGTCTCC, CTGTCTCT, CTGTGTCC, CTTCTCCC, TCCTCCCC, TCCTCTCC, TCCTGTCT, TCCTTCCT, TCTTCTCT, TGCTCTCC, TTCCTCCC, TTCTCCCT, TTCTCTCT, TTCTGCCT, TTGTCTCT, TTGTGTCT

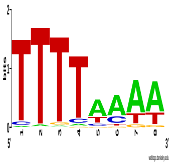

AAAAAGAA, AAAACAAA, TTCTAAAA, TTGTAAAA, TTGTTCTT, TTGTTTTT, TTTACAAA, TTTCCAAA, TTTTAAAA, TTTTAGAA, TTTTGAAA, TTTTGTCA, TTTTGTTT, TTTTTCTT, TTTTTTCA, TTTTTTTA, TTTTTTTT

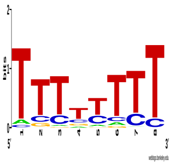

TCTCCTTT, TCTCTTTT, TCTGTTTT, TCTTCTTT, TCTTTTTT, TGTCCTTT, TGTGTTTT, TGTTCTTT, TGTTTTTT, TTATTTTT, TTCATTTT, TTCTCTTT, TTCTTTCT, TTCTTTTT, TTTATTCT, TTTATTTT, TTTCCATT, TTTCCTTT, TTTCTTTC, TTTCTTTT, TTTGCTTT, TTTGTTCT, TTTGTTTT, TTTTATTT, TTTTCTCT, TTTTCTTT, TTTTTATT, TTTTTGTT, TTTTTTCT, TTTTTTTC

1.2 Donor ISE elements

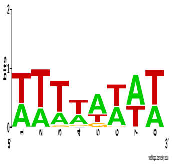

AAAAAAAA, AAAAATTT, AAAATTTT, AAATAAAA, AAATAAAT, AAATATAA, AAATATTT, AAATGAAA, AAATTTTT, AAGAAAAA, AAGAATTT, AAGATTTT, AATAAAAA, AATAATTT, AATTAAAA, AATTAAAT, AATTATTT, ATTAAAAA, ATTATTTT, ATTTAAAA, ATTTAAAT, ATTTATTT, TAAAAAAA, TTTAAAAA, TTTAAAAT, TTTAATAA, TTTAATTT, TTTATTAA, TTTATTAT, TTTATTTT, TTTCAAAA, TTTCAAAT, TTTCATTT, TTTGTTAT, TTTGTTTT, TTTTAAAA, TTTTAAAT, TTTTATAA, TTTTATTT, TTTTGAAA, TTTTGAAT, TTTTGTTT

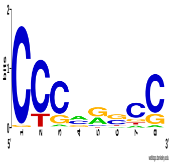

CCATTTCC, CCCACCCC, CCCACCTG, CCCACTCC, CCCAGAAA, CCCAGAAG, CCCAGACC, CCCAGAGC, CCCAGCCC, CCCAGCTG, CCCAGGAA, CCCAGGAG, CCCAGGCC, CCCAGGGC, CCCAGGTG, CCCCACCC, CCCCACTG, CCCCAGAG, CCCCAGCC, CCCCAGGC, CCCCTTCC, CCTCACCC, CCTCAGCC, CTGGGACC, CTGGGAGC, CTGGGCCC, CTGGGCTG, CTGGGGAA, CTGGGGAG, CTGGGGCC, CTGGGGGC, GTGGGGCC, GTGGGGGC

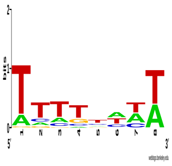

AAAAGAAA, AAAGAAAA, AAAGATTT, AAAGGAAA, AAGGAAAA, AAGGGAAA, AGAAGAAA, AGAGAAAA, AGAGGAAA, TCTAGAAA, TCTCTCTT, TCTCTTTT, TCTTCCTT, TCTTCTCT, TCTTCTTT, TCTTTCCT, TCTTTCTT, TCTTTGTT, TCTTTTCT, TCTTTTTT, TGAGGAAA, TTCCTTTT, TTCTCCTT, TTCTCTCT, TTCTCTTT, TTCTTAAA, TTCTTCCT, TTCTTCTT, TTCTTGTT, TTCTTTCT, TTCTTTTT, TTGGAAAA, TTTAGAAA, TTTCTAAA, TTTCTCCT, TTTCTCTT, TTTCTTCT, TTTCTTTT, TTTTCCCT, TTTTCCTT, TTTTCTCT, TTTTCTTT, TTTTTAAA, TTTTTCCT, TTTTTCTT, TTTTTGTT, TTTTTTCT, TTTTTTTT

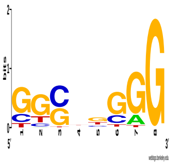

CAGGCGGG, CTCCTGGG, CTCTGCAG, CTCTGCTG, CTCTGGGG, CTCTGTGG, CTTCTGGG, GGCCTGGG, GGCTGCAG, GGCTGCTG, GGCTGGGG, GGCTGTGG, GGGAAAGG, GGGAACAG, GGGAAGGG, GGGAGCAG, GGGAGGGG, GGGAGTGG, GGGATGGG, GGGGCCAG,

GGGGCGGG, GGGTGCAG, GGTCTGGG, TCCCTGGG, TCCTGCAG, TCCTGCTG, TCCTGGGG, TCCTGTGG, TCTCTGGG, TTCCTGGG

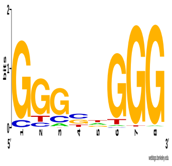

CCCCAGGG, CCCCTGGG, CCCTGGGG, CCTCTGGG, CTGCTGGG, CTGGAGGG, CTGGTGGG, CTGTGGGG, GCCCAGGG, GCCCTGGG, GCCTGGGG, GGAAGGGG, GGACAGGG, GGACTGGG, GGAGAGGG, GGAGCTGG, GGAGTGGG, GGCCAGGG, GGGCAGGG, GGGCTGGG, GGGGAGGG, GGGGCAGG, GGGGCCGG, GGGGCCTG, GGGGCTGG, GGGGTGGG, GGGTGGGG, GGGTGTGG, GTGCAGGG, GTGCTGGG, GTGGAGGG, GTGGTGGG, GTGTGGGG

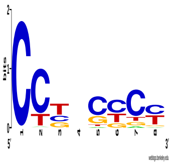

CCCAGCAC, CCCAGCCT, CCCAGCTC, CCCAGCTT, CCCAGGCA, CCCAGGCT, CCCTCCCC, CCCTCCCT, CCCTCTCC, CCCTCTCT, CCCTCTGC, CCCTCTGG, CCCTCTTC, CCTCCCCC, CCTCCCCT, CCTCCTCT, CCTCCTGC, CCTCCTGG, CCTCCTTC, CCTCTGCC, CCTCTGCT, CCTGAGCC, CCTGAGCT, CCTGCCAC, CCTGCCCC, CCTGCCCT, CCTGCCTC, CCTGCTCC, CCTGCTCT, CCTGCTGG, CCTGCTTC, CCTGGCAC, CCTGGCCC, CCTGGCCT, CCTGGCTC, CCTGGGCC, CCTGGGCT, CCTTCCCC, CCTTCCCT, CCTTCCTC, CCTTCTCT, CCTTCTGC, CCTTCTGG, CCTTCTTC, CTGCCCAC, CTGCCCCA, CTGCCCCC, CTGCCCCT, CTGCCCTC, CTGCCTCC, CTGCCTCT, CTGCCTGC, CTGCCTGG, CTGCCTTC, CTGCTCCC, CTGCTGCC, CTGCTGCT

1.3 ESE elements

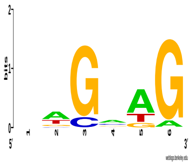

AAGGAG, ATGAAG, ATGCTG, ATGGAG, CTGAAA, GAGAAA, GAGAAG, GAGGAA, GAGGAG, GCCTGG, TGGAAG, TGGCTG, TGGGAG

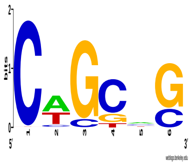

CAGCCC, CAGGAG, CTGCCC

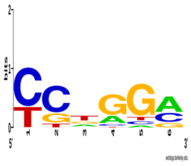

CCAGCC, CCAGGC, CCATCC, CCTGCC, CCTGGC, TGGAGC, TGGATG, TTCCAG, TTCCTG, TTTGGA, TTTGGC

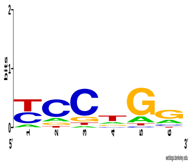

ACCAGG, ACCTGC, ACCTGG, ATGCAA, ATGCTT, ATGGCA, CAAGAC, CAAGCA, CAGCTT, CAGGAA, CAGGCA, CATCAC, CATCTC, CATCTT, CCCAGC, CCCTGC, TCAGCA, TCCAGA, TCCAGC, TCCAGG, TCCTGA, TCCTGC, TCCTGG, TCCTGT, TGCAGG, TGCTGT, TGGGAA

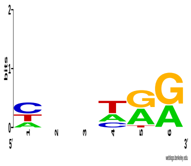

AAGAAA, AAGAAG, AAGATG, CAGAAG, CCCTGA, CCCTGG, CTTCAG, CTTTGA, CTTTGG, TGATGA, TGATGG, TGCTGA

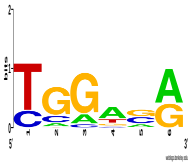

CAGACA, CAGAGG, CAGTGG, CCACCA, CCAGCA, TGCTGG, TGGACA, TGGAGG, TGGCCA, TGGTGG, TTCTGG

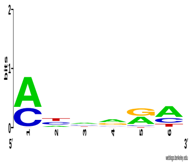

AAAAGA, AAAAGG, AAAAGT, AAACAA, AAACAC, AAAGAC, AAAGTA, ACCAGA, ACCAGT, AGCAGA, AGCAGG, AGCAGT, ATCAGA, ATCAGT, ATGGAA, ATGGAC, ATGGTA, ATTCAA, ATTCAC, ATTGAA, ATTGAC, ATTGTA, ATTTCC, ATTTCT, ATTTGA, CCAAGA, CCAAGG, CCAAGT, CCAGAC, CCAGTA,

CCCAGG, CCCAGT, CCCCAA, CCCCAC, CCGGAA, CCGGAC, CCGGTA, CCTCAA, CCTCAC, CCTTCC, CCTTCT, CCTTGA, CTCAGA, CTGGAC, CTTCAA, CTTCAC, CTTTCC, CTTTCT, TGCAGA

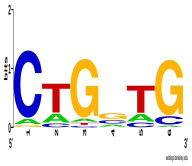

AAGCTG, AAGGTG, ATGCAG, ATGGTG, CAAGTG, CAGATG, CAGCAG, CAGCTG, CAGGCC, CAGGTG, CCAGTG, CCCATG, CCTCAG, CCTGTG, CTGAAG, CTGACC, CTGATG, CTGCAA, CTGCAG, CTGGAG, CTGGCC, CTGGTG, CTGTCC, CTGTTG, GAGATG, GAGCAG, GAGCTG, GAGGTG

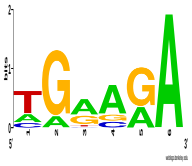

AGAAAA, AGAAGA, AGACAA, AGGAAA, AGGAGA, AGGCAA, CACAGA, CACCAA, CAGAAA, CAGAGA, CATCAA, CATGAA, CATGGA, TGAAAA, TGAAGA, TGACAA, TGAGAA, TGAGGA, TGGAAA, TGGAGA, TGGCAA

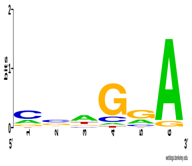

AAAGAA, AAAGGA, AGAGAA, AGAGGA, CAAGAA, CAAGGA, CCAGAA, CCAGGA, CCCAAA, CCCACA, CCCAGA, CCCTCA, CCTCCA, CCTCTG, CCTGAA, CCTGGA, CTGCCA, CTGCTG, CTGGAA, CTGGGA, GGAGAA, GGAGGA, TCCTCA, TCTGGA, TTGCCA, TTGCTG, TTGGGA
